# Convolutional neural network-based regression analysis to predict subnuclear chromatin organization from two-dimensional optical scattering signals

**DOI:** 10.1117/1.JBO.29.8.080502

**Published:** 2024-08-28

**Authors:** Yazdan Al-Kurdi, Cem Direkoǧlu, Meryem Erbilek, Dizem Arifler

**Affiliations:** a Middle East Technical University, Northern Cyprus Campus, Electrical and Electronics Engineering Program, Kalkanli, Turkey; b Middle East Technical University, Northern Cyprus Campus, Computer Engineering Program, Kalkanli, Turkey; c Middle East Technical University, Northern Cyprus Campus, Physics Group, Kalkanli, Turkey

**Keywords:** chromatin organization, convolutional neural network, finite-difference time-domain modeling, machine learning, optical scattering, regression analysis

## Abstract

**Significance:**

Azimuth-resolved optical scattering signals obtained from cell nuclei are sensitive to changes in their internal refractive index profile. These two-dimensional signals can therefore offer significant insights into chromatin organization.

**Aim:**

We aim to determine whether two-dimensional scattering signals can be used in an inverse scheme to extract the spatial correlation length $\ell_{c}$ and extent δn of subnuclear refractive index fluctuations to provide quantitative information on chromatin distribution.

**Approach:**

Since an analytical formulation that links azimuth-resolved signals to $\ell_{c}$ and δn is not feasible, we set out to assess the potential of machine learning to predict these parameters via a data-driven approach. We carry out a convolutional neural network (CNN)-based regression analysis on 198 numerically computed signals for nuclear models constructed with $\ell_{c}$ varying in steps of 0.1  μm between 0.4 and 1.0  μm, and δn varying in steps of 0.005 between 0.005 and 0.035. We quantify the performance of our analysis using a five-fold cross-validation technique.

**Results:**

The results show agreement between the true and predicted values for both $\ell_{c}$ and δn, with mean absolute percent errors of 8.5% and 13.5%, respectively. These errors are smaller than the minimum percent increment between successive values for respective parameters characterizing the constructed models and thus signify an extremely good prediction performance over the range of interest.

**Conclusions:**

Our results reveal that CNN-based regression can be a powerful approach for exploiting the information content of two-dimensional optical scattering signals and hence monitoring chromatin organization in a quantitative manner.

## 1 Introduction

It is well established that optical scattering signals are sensitive to alterations in the morphology and internal structure of tissue constituents. This has paved the way for the development of numerous optical tools that can be employed to monitor cells or cell nuclei, mitochondria, lysosomes, and fibrous networks for various diagnostic purposes.^1^^,^^2^ Cell nuclei serve as repositories of genetic material and have thus far been a major focus for the diagnosis of diseases. In particular, changes in chromatin organization within cell nuclei are important indicators of precancer progression. Previous studies provide strong evidence that such changes can be detected via scattering-based optical modalities.^3^^–^^14^

Subnuclear chromatin distribution can be modeled as a continuum of refractive index fluctuations.^15^ For a quantitative description, one parameter of interest is functional factor D, which controls the shape of the refractive index correlation function. This parameter is related to chromatin packing scaling and is a measure of the heterogeneity of chromatin distribution. Two other parameters of interest are the characteristic length scale of refractive index fluctuations and the extent of refractive index fluctuations, which together allow for a more intuitive characterization of chromatin organization. The length scale of fluctuations represents the characteristic size of subnuclear structures and can be defined in terms of the spatial correlation length $\ell_{c}$, roughly indicating the distance over which the correlation of refractive index values drops to a negligible level. The extent of fluctuations, on the other hand, can be defined as the standard deviation δn of refractive index values within the nucleus and is directly related to the inhomogeneity of macromolecular density. These two parameters are often lumped into a single quantity referred to as the disorder strength and expressed as $L_{d}=\ell_{c}\delta n^{\alpha }$, where α is usually set to 1 or 2.^3^^,^^4^^,^^9^^,^^14^^,^^16^ It is possible to extract information on D, $L_{d}$, or both using scattering-based optical techniques along with relevant analytical formulations of light propagation or algorithms to analyze measurements. In fact, a number of prior studies on low-coherence enhanced backscattering spectroscopy, inverse spectroscopic optical coherence tomography, partial-wave spectroscopic microscopy, or quantitative phase imaging show that nuclear D and $L_{d}$ tend to increase with the progression of cancer.^3^^–^^5^^,^^8^^,^^13^^,^^14^ This most likely corresponds to chromatin compaction that is expected to manifest as an increase in heterogeneity of subnuclear chromatin organization. Reporting on $\ell_{c}$ and δn separately as in Refs. 6 and 10 can possibly be more informative since independent assessment of changes in both parameters can lead to a more direct interpretation of alterations in chromatin distribution.

We recently demonstrated that azimuth-resolved optical scattering signals obtained from cell nuclei can provide significant insights into their internal refractive index profile.^11^ Features calculated based on azimuth-dependent intensity variations in these two-dimensional signals are sensitive to the length scale and extent of subnuclear refractive index fluctuations; further, these features are not susceptible to changes in the overall size, shape, and mean refractive index of nuclei. Therefore, our results indicate that precancer-related changes in chromatin organization can be selectively monitored via analysis of two-dimensional scattering signals.

An important question that arises is whether we can use two-dimensional scattering signals in an inverse scheme to extract the spatial correlation length $\ell_{c}$ and extent δn of refractive index fluctuations to obtain a quantitative measure of subnuclear chromatin distribution. Since an analytical formulation that links azimuth-resolved signals to $\ell_{c}$ and δn is not feasible, it is best to resort to a data-driven approach. Machine learning and deep learning methods are increasingly being used in the field of biomedical optics. These methods have also been applied to scattering-based measurements, yet mainly for classification purposes.^12^^,^^13^^,^^17^^–^^23^ In this work, we present a convolutional neural network (CNN)-based regression analysis aimed at the extraction of $\ell_{c}$ and δn from two-dimensional scattering signals. Our dataset consists of numerically computed signals for three-dimensional nuclear models constructed with varying values of $\ell_{c}$ and δn. The results obtained with this dataset show that CNN-based regression on scattering signals provides a potential means to extract both parameters and make predictions on subnuclear chromatin organization.

## 2 Methods

### 2.1 Two-Dimensional Optical Scattering Signals

The methodology for obtaining the set of optical scattering signals used in the study presented here has been previously described.^11^ Briefly, nuclear models were constructed in voxelated grids as spheres with a radius of 4.0  μm or ellipsoids with semiaxis lengths of 3.0  μm, 4.0  μm, and 5.0  μm. The mean refractive index of the nuclei was set to 1.40, and the refractive index of the embedding cytoplasm was assumed to be 1.36. A stochastic approach was adopted to generate subnuclear refractive index fluctuations; the spatial refractive index profile conformed to a Gaussian correlation function. The values of the correlation length $\ell_{c}$ were selected from {0.4, 0.5, 0.6, 0.7, 0.8, 0.9, 1.0} μm, and the values of the extent δn of refractive index fluctuations were selected from {0.005, 0.010, 0.015, 0.020, 0.025, 0.030, 0.035}. Three different nuclear models were constructed for each combination of $\ell_{c}$ and δn considered. Overall, there were a total of 198 models (including 99 spherical and 99 ellipsoidal models) available for our study. Each constructed nuclear model was fed as input into an in-house finite-difference time-domain (FDTD) simulation code to numerically compute the resulting optical scattering response at a wavelength of 800 nm. The simulation output consisted of a two-dimensional array of scattered light intensities I(θ,ϕ), where θ∈{0,1,…,180} was the polar scattering angle, defined to be the angle between the incident and scattered light directions, and ϕ∈{0,1,…,360} was the azimuthal scattering angle, defined to be the angle between the incident wave polarization direction and the scattering plane, both in degrees. Note that each simulation output I(θ,ϕ) covers a dynamic range of $∼10^{10}$. The two-dimensional FDTD signals presented henceforth and employed for analysis correspond to $\log_{10}[I(\theta ,\phi )]$ subjected to a full-scale contrast stretch algorithm to produce image-like functions denoted by $\overset{˜}{I}(\theta ,\phi )$ with values varying between 0 and 255.

**Fig. 1:**
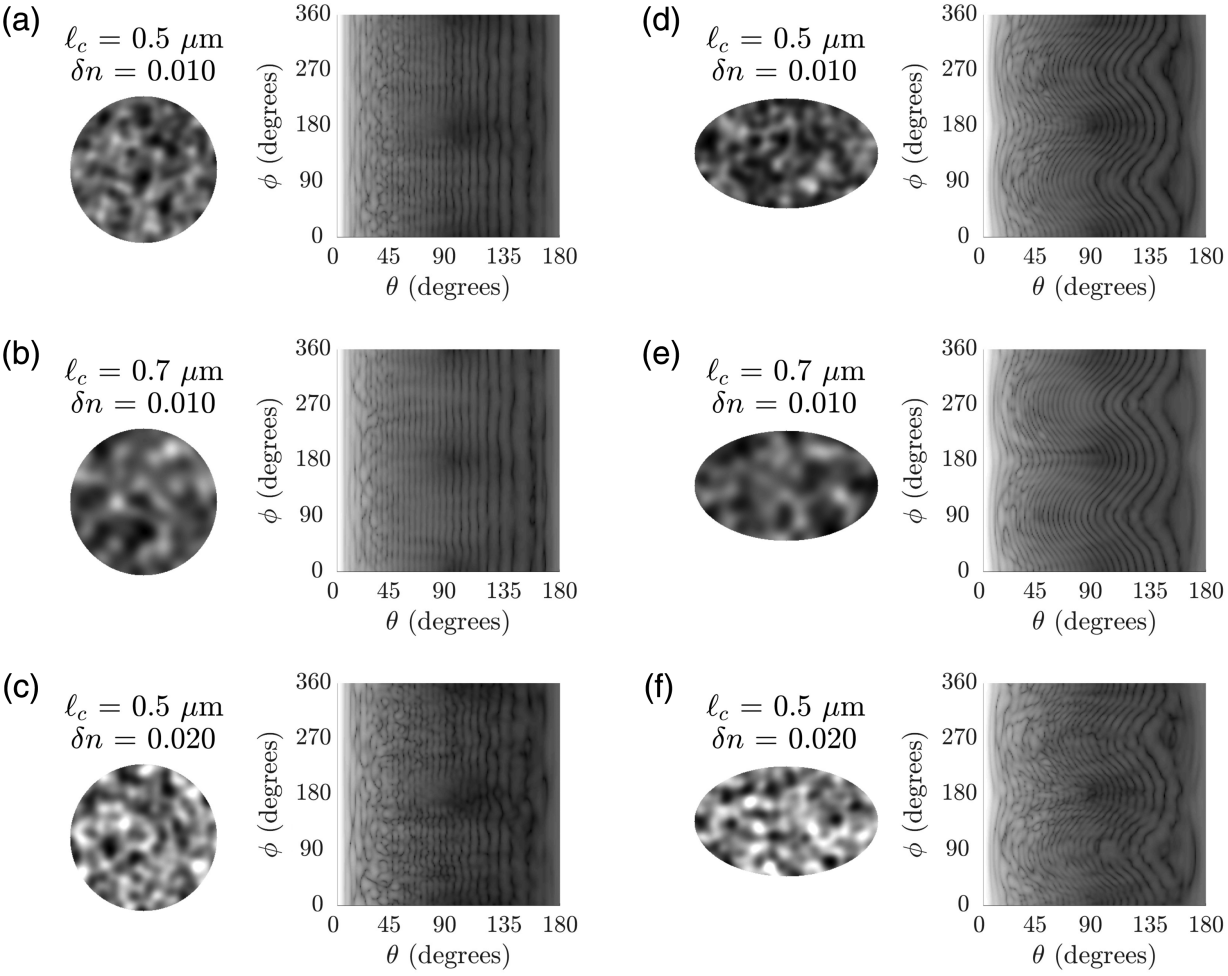
Central cross-sections from sample constructed nuclear models and the corresponding two-dimensional optical signals obtained via FDTD simulations. The mean refractive index of the models is 1.40 and the refractive index of the embedding cytoplasm is 1.36 in all cases. Spherical models have a radius of 4.0  μm with refractive index fluctuations characterized by (a) $\ell_{c}=0.5\;\mu m$, δn=0.010; (b) $\ell_{c}=0.7\;\mu m$, δn=0.010; and (c) $\ell_{c}=0.5\;\mu m$, δn=0.020. Ellipsoidal models have semiaxis lengths of 3.0  μm, 4.0  μm, and 5.0  μm with refractive index fluctuations characterized by (d) $\ell_{c}=0.5\;\mu m$, δn=0.010; (e) $\ell_{c}=0.7\;\mu m$, δn=0.010; and (f) $\ell_{c}=0.5\;\mu m$, δn=0.020. The grayscale for depiction of nuclear cross-sections is adjusted so that darker areas correspond to regions of higher refractive index. The FDTD signals are rescaled to have values varying between 0 and 255.

Figure 1 shows sample FDTD signals obtained for spherical or ellipsoidal nuclear models with different values of $\ell_{c}$ and δn. Depictions of central cross-sections of the constructed models are also included for reference; the grayscale for these cross-sectional depictions is adjusted so that darker areas correspond to regions of higher refractive index. As discussed in detail in Ref. 11, the signals for the spherical nuclear models are characterized by vertical background fringes [Figs. 1(a)–1(c)], whereas the signals for the ellipsoidal nuclear models are characterized by curved background fringes [Figs. 1(d)–1(f)]. In both cases, however, the signals become more irregular with significant intensity variations along the ϕ direction when $\ell_{c}$ decreases or when δn increases.

### 2.2 CNN-Based Regression Analysis

**Fig. 2:**
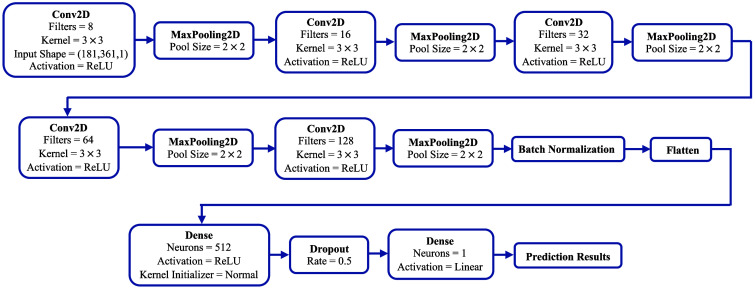
Block diagram of our CNN architecture used for regression analysis on two-dimensional optical scattering signals corresponding to nuclear models with varying internal refractive index profiles. Prediction results include the correlation length $\ell_{c}$ and extent δn of nuclear refractive index fluctuations.

A convolutional neural network (CNN) is a type of deep neural network primarily applied for image classification and regression tasks. We followed standard CNN frameworks^24^^–^^26^ and designed an architecture for the regression task at hand to be able to predict the correlation length $\ell_{c}$ and extent δn of nuclear refractive index fluctuations from two-dimensional optical scattering signals. We implemented our design in Google Colab using the TensorFlow library.^27^ The details regarding our CNN architecture are provided in the block diagram in Fig. 2. As described in Section 2.1, input signals had dimensions of 181×361. We used five convolutional (Conv) layers with the ReLU activation function; these Conv layers, from the first to the fifth layers, had 8, 16, 32, 64, and 128 filters of size 3×3, respectively. After each Conv operation, we applied the MaxPooling operation with a pool size of 2×2. At the end of the fifth Conv layer, batch normalization was performed and data was flattened so that it could be input to a Dense layer with 512 neurons and the ReLU activation function. We also had a dropout operation after the Dense layer with a rate of 0.5; dropout is a technique to prevent overfitting, and a rate of 0.5 is typically observed to be effective in improving the generalization performance for a wide range of networks and tasks.^25^^,^^28^ The final layer had only one neuron with the linear activation function to give the prediction results. We used the Adam optimizer,^29^ which is an algorithm for the first-order gradient-based optimization of stochastic objective functions. This algorithm relies on adaptive estimates of lower-order moments and combines the advantages of two other common optimizers, namely AdaGrad and RMSProp; it is one of the most popular methods for training as it is computationally efficient, has low memory requirements, and needs minimal hyperparameter tuning. We selected and tuned our hyperparameters as suggested in Ref. 29: the step size or learning rate α was set to 0.0005; the exponential decay rates $\beta_{1}$ and $\beta_{2}$ for the first- and second-moment estimates were set to 0.9 and 0.999, respectively; and ε, a small constant to prevent division by zero, was assigned a value of $10^{-7}$. A callback function, which saved the weights that gave the lowest validation loss value, was also employed. We note that our dataset, consisting of 198 signals, was randomly partitioned into training, validation, and test subsets with split ratios of 60%, 20%, and 20%, respectively. Five-fold cross-validation was used to assess the prediction performance of the CNN.

## 3 Results and Discussion

**Fig. 3:**
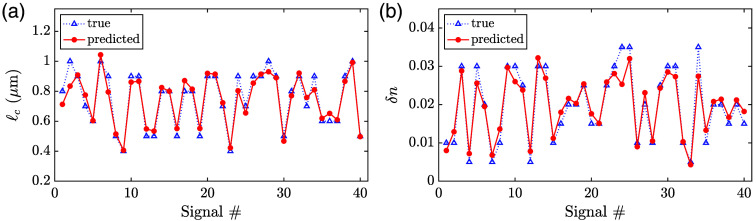
Prediction results for (a) $\ell_{c}$ and (b) δn, both obtained for the same 40 signals in a single test set. The triangular (Δ) markers represent the true values, and the circular (•) markers represent the predicted values.

**Fig. 4:**
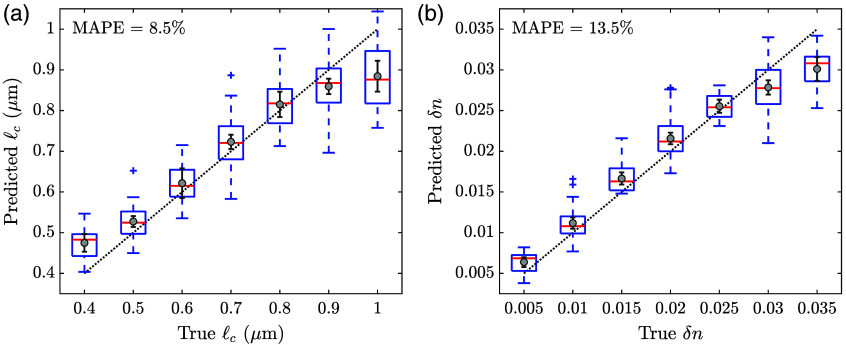
Overall assessment of prediction results for (a) $\ell_{c}$ and (b) δn. The central marks in the box plots show the median predicted values, the bottom and top edges of the boxes indicate the 25th and 75th percentiles, the whiskers extend to the most extreme values that are not considered outliers, and the outliers are plotted individually using plus (+) markers. The circular (o) markers in the plots correspond to the mean values of the predicted values, with error bars indicating 95% confidence intervals of the mean values. The dotted diagonal lines in the background represent perfect agreement. The mean absolute percent errors (MAPEs) for $\ell_{c}$ and δn are 8.5% and 13.5%, respectively.

Figure 3 shows the prediction results for 40 signals in a single test set. The triangular markers represent the true values, and the circular markers represent the predicted values. For this set, we observe a close agreement between the true and predicted values for both $\ell_{c}$ [Fig. 3(a)] and δn [Fig. 3(b)]. Similar results are observed for the other test sets. To illustrate and quantify the overall performance of our CNN-based regression analysis, we combine the results obtained for all test sets in Fig. 4, and we compute the mean absolute percent errors (MAPEs) for both parameters. The central marks in the box plots for $\ell_{c}$ [Fig. 4(a)] and δn [Fig. 4(b)] show the median predicted values, and the bottom and top edges of the boxes indicate the 25th and 75th percentiles, respectively. The whiskers extend to the most extreme values that are not considered outliers, and the outliers are plotted individually using plus markers. The circular markers in the plots correspond to the mean values of the predicted values, with error bars indicating 95% confidence intervals of the mean values. Note that the dotted diagonal lines in the background represent perfect agreement and are meant to guide the eye. The MAPEs computed for $\ell_{c}$ and δn are 8.5% and 13.5%, respectively. Since our nuclear models were constructed such that $\ell_{c}$ varied in steps of 0.1  μm between 0.4 and 1.0  μm, and δn varied in steps of 0.005 between 0.005 and 0.035, these errors are smaller than the minimum percent increment between successive values for respective parameters and can thus be considered to signify an extremely good prediction performance over the range of interest. Further, the MAPEs that we obtained here are comparable to those previously reported in a similar context.^10^

It is important to point out that an increase in $\ell_{c}$ and a decrease in δn give rise to similar changes in two-dimensional scattering signals [Figs. 1(a)–1(f)]. Hence, isolating the effects of these two parameters is quite challenging. Our results reveal that a CNN-based approach can be used to tackle this issue; close agreement between the true and predicted values suggests that both parameters can be independently quantified. This definitely proves advantageous because mutually exclusive information on the length scale and extent of refractive index fluctuations can provide more specific details regarding the internal structure of the cell nuclei. It is also worth reiterating that our dataset included optical scattering signals from both spherical and ellipsoidal nuclear models. We can thus claim that refractive index fluctuations can be well characterized despite shape-dependent differences in signals, as observed between Figs. 1(a)–1(c) and Figs. 1(d)–1(f).

The results presented here, albeit obtained with a limited dataset, offer strong evidence that CNN-based regression can be a powerful approach to exploit the information content of two-dimensional optical scattering signals obtained from cell nuclei and to monitor chromatin organization in a quantitative manner. Analysis on an extended dataset will potentially lead to a broader perspective on how to refine the CNN architecture for an improved performance. It is apparent from Figs. 3 and 4 that the most notable deviations between the true and CNN-predicted values are observed for large $\ell_{c}$ or large δn. We presume that a relatively smaller number of nuclear models constructed with large $\ell_{c}$ and large δn is the main underlying factor for this trend. As such, training with an extended dataset that includes more cases with large parameter values is likely to result in a better prediction performance.

From a practical perspective, experimental setups for the acquisition of two-dimensional optical scattering signals from cells have already been implemented and described in a series of studies.^17^^–^^21^^,^^30^^,^^31^ The specific angular ranges for signal acquisition vary depending on the particular setup employed, but it is common to see a wide range of side scattering covered with an angular sampling interval of one degree or less. In fact, we previously reported that azimuth-dependent intensity variations in scattering signals over the angular range of θ=40−140  deg are extremely sensitive to subnuclear refractive index fluctuations.^11^ Hence, limiting the range of interest in a CNN-based regression analysis to side scattering angles may even aid in improving the prediction performance, leading to smaller MAPEs. In addition, this will have the added benefit of reducing the dynamic range of optical scattering signals to be measured to $∼10^{5}$ or $∼10^{4}$. CNN algorithms can actually be combined with optimization routines to determine the optimal angular range and angular sampling interval that can be used to minimize MAPEs so that scanning and detection systems can be designed and fine-tuned accordingly.

On a related matter, we also note that a full assessment of our analysis approach requires a detailed characterization of any influence of noise that will inevitably be present in real measurements. To offer some preliminary insights into the potential influence of noise on the prediction performance, we added zero-mean white Gaussian noise^32^^,^^33^ to the two-dimensional optical scattering signals in the test sets and applied our CNN algorithm trained with noise-free signals to noise-degraded signals. Here, the noise level was quantified in terms of the signal-to-noise ratio given by $\mathrm{SNR}=10\;\log_{10}(S/N)$, where $S=\Sigma_{\theta ,\phi }[\tilde{I}(\theta ,\phi ){]}^{2}/(181\times 361)$ represents the average signal power and N represents the average noise power, which is equal to the Gaussian noise variance. For an SNR of 25 dB, roughly corresponding to the case in which the noise standard deviation is 5% of the root-mean-square signal value in line with Ref. 34, the MAPEs obtained for $\ell_{c}$ and δn are 12.3% and 14.1%, respectively. These results do not point to a significant decrease in prediction performance for the noise level specified. This can be regarded as initial evidence that our CNN-based regression algorithm trained with simulated data can possibly be applied to real measurements; that said, the corroboration of predicted values via high-resolution imaging techniques will be the ultimate benchmark. A comprehensive analysis of performance deterioration due to higher noise levels will certainly provide guidelines for devising noise reduction strategies that should be explored as part of any study involving an inverse scheme for prediction of relevant parameters from measurements.

In our study, we assumed that the main contribution to cellular scattering comes from the nucleus. Hence, our nuclear models were constructed in a homogeneous cytoplasm with a fixed refractive index. We remark that this is a valid assumption for cells that are characterized by a very low volume fraction of organelles.^8^^,^^11^ For cells with a high volume fraction of organelles, however, we cannot exclude contributions from the cytoplasm. In that case, we need to assess the influence of mitochondria or other subcellular structures on two-dimensional scattering signals. There is also a need to determine whether surface roughness as discussed in Refs. 21 and 35 can be a compounding factor for the prediction of parameters related to the subnuclear refractive index profile. A systematic investigation based on numerical studies as presented in this work will potentially reveal whether a CNN-based analysis can distinctively pick out features linked to different sources of scattering. We intend to address these issues as part of our future research efforts.

## 4 Conclusions

In summary, the research described here highlights the potential of CNN-based regression on two-dimensional optical scattering signals obtained from cell nuclei to extract the length scale and extent of internal refractive index fluctuations. Even though our work focuses on the prediction of chromatin organization, which is strongly linked to precancer progression, a similar methodology can be used to monitor the internal refractive index profiles of other subcellular organelles or tissue constituents. This can facilitate scattering-based delineation of the progressive development of a wide spectrum of diseases.

## Data Availability

The set of simulated optical signals used in this letter is available from the corresponding author upon reasonable request.
